# Intracellular B Lymphocyte Signalling and the Regulation of Humoral Immunity and Autoimmunity

**DOI:** 10.1007/s12016-017-8609-4

**Published:** 2017-04-29

**Authors:** Taher E. Taher, Jonas Bystrom, Voon H. Ong, David A. Isenberg, Yves Renaudineau, David J. Abraham, Rizgar A. Mageed

**Affiliations:** 1 Centre for Experimental Medicine and Rheumatology, William Harvey Research Institute, Queen Mary University of London, Charterhouse Square, London, EC1M 6BQ UK; 2 Centre for Rheumatology and Connective Tissue Diseases, Royal Free Hospital, University College London, London, UK; 3 Centre for Rheumatology, University College London, London, UK; 4 Immunology Laboratory, University of Brest Medical School, Brest, France

**Keywords:** B lymphocytes, Intracellular signalling, Autoimmune diseases

## Abstract

B lymphocytes are critical for effective immunity; they produce antibodies and cytokines, present antigens to T lymphocytes and regulate immune responses. However, because of the inherent randomness in the process of generating their vast repertoire of antigen-specific receptors, B cells can also cause diseases through recognizing and reacting to self. Therefore, B lymphocyte selection and responses require tight regulation at multiple levels and at all stages of their development and activation to avoid diseases. Indeed, newly generated B lymphocytes undergo rigorous tolerance mechanisms in the bone marrow and, subsequently, in the periphery after their migration. Furthermore, activation of mature B cells is regulated through controlled expression of co-stimulatory receptors and intracellular signalling thresholds. All these regulatory events determine whether and how B lymphocytes respond to antigens, by undergoing apoptosis or proliferation. However, defects that alter regulated co-stimulatory receptor expression or intracellular signalling thresholds can lead to diseases. For example, autoimmune diseases can result from altered regulation of B cell responses leading to the emergence of high-affinity autoreactive B cells, autoantibody production and tissue damage. The exact cause(s) of defective B cell responses in autoimmune diseases remains unknown. However, there is evidence that defects or mutations in genes that encode individual intracellular signalling proteins lead to autoimmune diseases, thus confirming that defects in intracellular pathways mediate autoimmune diseases. This review provides a synopsis of current knowledge of signalling proteins and pathways that regulate B lymphocyte responses and how defects in these could promote autoimmune diseases. Most of the evidence comes from studies of mouse models of disease and from genetically engineered mice. Some, however, also come from studying B lymphocytes from patients and from genome-wide association studies. Defining proteins and signalling pathways that underpin atypical B cell response in diseases will help in understanding disease mechanisms and provide new therapeutic avenues for precision therapy.

## Introduction

**Fig. 1:**
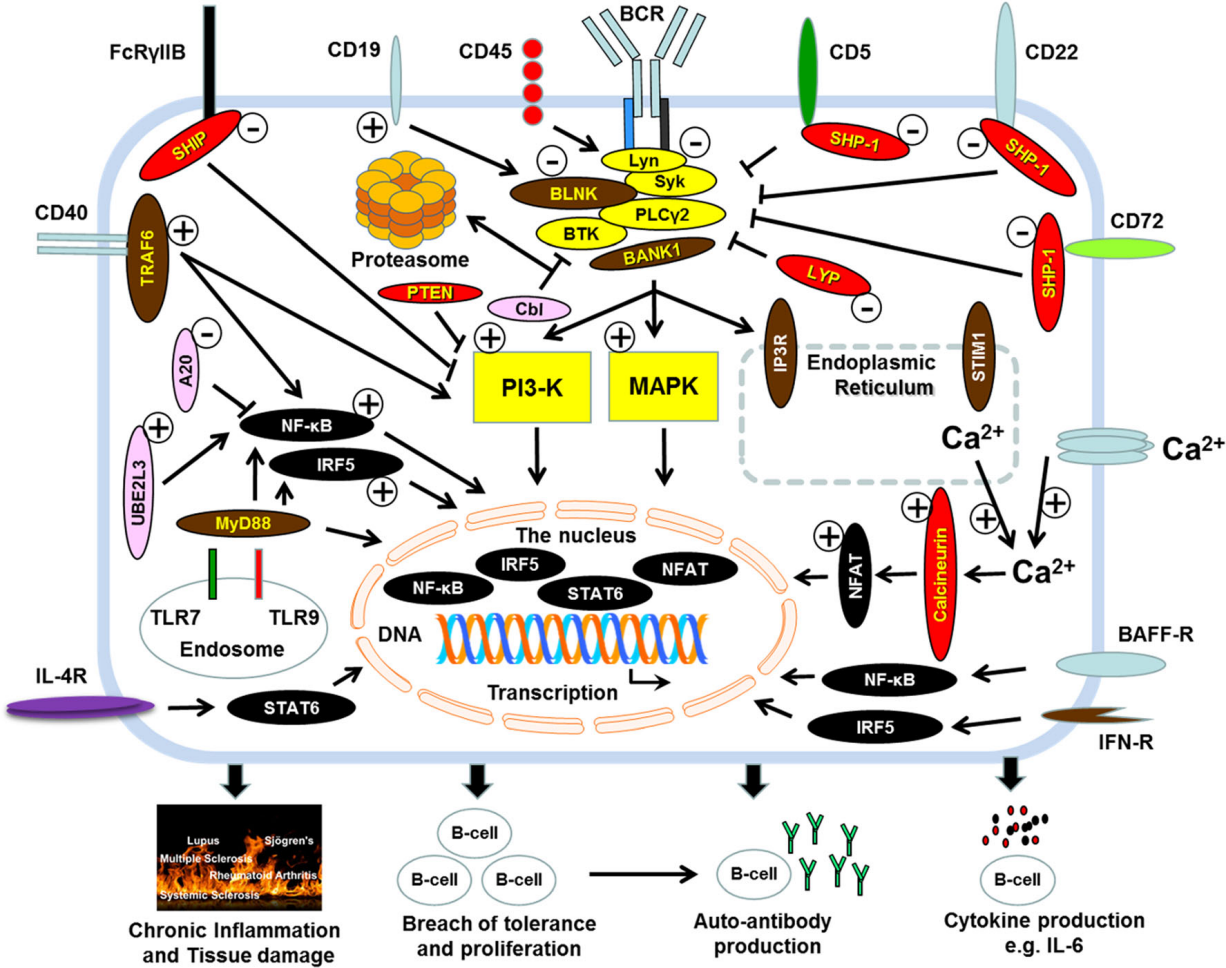
Signalling molecules and pathways in regulating B cell selection and responses. The diagram illustrates major signalling proteins/pathways involved in B cell physiology and whose regulation has been reported to be altered/defective in B cells in autoimmune disease. Proteins indicated in *yellow* are kinases, *red* for phosphatases, *pink* for proteins involved in ubiquitination, *black* for transcription factors and *brown* for adaptor proteins. *Arrows* indicate proteins that promote positive signalling, while *blunt-ended lines* indicate the protein negatively regulate signalling. *Minus signs* (*encircled*) indicate proteins/signalling pathways are reduced in mice and/or patients with autoimmune diseases or that reduction by genetic engineering promotes B lymphocyte hyperactivity and autoimmune disease. *Positive signs* (*encircled*) indicate enhanced activity of the proteins/signalling pathways in B lymphocytes from patients with autoimmune disease, mouse models or that their genetic manipulation promotes autoimmunity

Autoimmune diseases are pathological conditions in which defects in immunological tolerance to self lead to the initiation of effector immunity to self, chronic inflammation and tissue and organ damage. These diseases affect about 5–10% of human populations worldwide and cause significant degrees of morbidity and early death [1]. The cause of most autoimmune diseases remains largely unknown. However, susceptibility to develop these diseases is associated with a combination of genetic, environmental and hormonal factors [2]. These factors combine to cause defects in the survival and selection of self-reactive T and B lymphocytes. Evidence from the last 50 years of research indicates that T lymphocytes initiate autoimmune responses in conjunction with, or following incitement by, B lymphocytes. In addition to autoantibody production, there is compelling evidence that B lymphocytes also contribute to the development of the autoimmune diseases through mechanisms such as autoantigen presentation to activate autoreactive T cells and/or promote their polarization to produce disease-promoting/perpetuating cytokines. In this respect, it is perhaps revealing that a significant proportion of genetic susceptibility risk factors to develop autoimmune diseases corresponds with defects in the regulation of B cell responses, intracellular signalling and tolerance induction. These observations highlight changing perceptions about the role played by B cells in autoimmune diseases (Fig. 1; Tables 1 and 2). The importance of these roles is supported by the therapeutic benefit gained from depleting B cells in patients with a range of autoimmune diseases. For example, patients with diseases such rheumatoid arthritis (RA) [71], type 1 diabetes (T1D) [72], anti-neutrophil cytoplasmic antibody (ANCA) vasculitis [73], multiple sclerosis (MS) [74], systemic sclerosis (SSc) [75, 76], primary Sjögren’s syndrome [77–80] and systemic lupus erythematosus (SLE) [81–83] benefit from therapeutic depletion of B cells. Of note in this respect is that B cell-depleting therapy has a clinical benefit without significantly affecting autoantibody levels, suggesting that, perhaps, other B cell functions, including antigen presentation and cytokine production could be critical aspects of B cell involvement in the pathogenesis of the autoimmune diseases.

**Table 1:** Reported defects in signalling molecules and pathways and their impact on B cell responses and association with diseases

Signalling molecule	Encoding gene	Effect on B cell response and disease in animal models	Association with human diseases
Lyn	*LYN*	B cell hyperactivity causing lupus-like disease in gene-deficient mice [3]	Reduced cellular expression leading to IgG autoantibody and cytokine production [4]
SHP-1	*PTPN6*	Selective deficiency in B cells promotes systemic autoimmune disease [5]	Reduced cellular expression and SNP association with SLE [6, 7]
LYP	*PTPN22*	Expression of the R619W variant in B cells causes systemic autoimmunity [8]	The R619W is a risk allele in several systemic autoimmune diseases [9, 10]
CD45	*CD45*	Mutation in the inhibitory wedge causes autoantibody production leading to severe glomerulonephritis [11]	Decreased expression, increased translocation signalling domains and altered isoform expression associated with SLE [12]
BTK	*BTK*	Over expression increases plasma cell numbers, spontaneous germinal centre formation, autoantibody production and lupus-like disease [13, 14]	Gene defect causes X-linked agammaglobulinemia, reduced B cell numbers and deficiency in all immunoglobulin isotypes [15]. Increased phosphorylation in SLE B cells [16]
CD22	*CD22*	Deficiency causes autoantibody production and lupus-like disease [17, 18]	Splicing defect causes expression of a truncated CD22 expression and increased leukemic B cell precursors [19]
CD19	*CD19*	Altered expression correlates with autoimmune diseases [20, 21]	Increased expression in patients with systemic sclerosis; polymorphism is associated with susceptibility to SLE [22, 23]
FCγRIIB	*FCγRIIB*	Deficiency causes SLE-like autoimmune disease and renders non-permissive H2B mouse strain susceptible to collagen-induced arthritis (CIA) [24, 25]	Decreased expression in SLE [26]
SHIP-1	*INPP5D*	B cell-specific deficiency causes lupus-like disease [27]	Hypophosphorylated in SLE patients [28]
PTEN	*PTEN*	B cell-specific deficiency causes hyperresponsiveness and anti-ssDNA autoantibody production [29, 30]	Decreased expression in SLE patients [31]
PTP1B	*PTPN1*	B cell-specific deficiency causes systemic autoimmunity in aged mice [32]	Reduced expression in RA patients [32]
Act1	*TRAF3IP2*	Deficient mice develop Sjögren’s syndrome-like disease [33]	Susceptibility gene in psoriatic arthritis and SLE and SNP associated with RA [34–36]
A20	*TNFAIP3*	B cell-specific deficiency causes systemic autoimmunity [37–39]	SNPs associated with SLE and RA [40, 41]
Cbl	*CBL*	B cell-specific deficiency of c-Cbl and Cbl-b causes systemic autoimmunity [42]	SNP associated with SLE and type 1 diabetes [43, 44]
WASP	*WAS*	B cell-specific deficiency causes systemic autoimmune disease [45]	About 40% of Wiskott-Aldrich syndrome patients develop autoimmunity [46]

The table summarizes data on reported clinical and immunological phenotypes in engineered mice gene deficient/mutated for signalling proteins. The table also provides some of the reported data on defects in the expression or function of the corresponding protein in patients

**Table 2:** Polymorphisms and mutations in genes encoding co-receptors, signalling proteins, transcription factors and cytokines/chemokines that are associated with human diseases

Gene	Chromosome	Disease association	Protein	Function in B cells	Reference
*PTPN22*	1p13.2	RA, SLE, GT, T1D	LYP	Lymphocyte-specific tyrosine phosphatase^a^	[47]
*NCF2*	1q25	SLE	p67phox	Subcomponent of NADPH oxidase, ROS generation^a^	[48]
*IL10*	1q31-q32	SLE, UC, T1D	IL-10	Anti-inflammatory cytokine^a^	[49]
*PTPRC*	1q31.3-q32.1	SLE, RA, MS, T1D	CD45	Membrane protein tyrosine kinases	[50]
*FCGR2A*	1q23.2	SLE, RA	IGFR2	Low-affinity IgG FC receptor^a^	[51, 52]
*RASGRP3*	2p25.1-p24.1	SLE	GRP3	Signalling downstream of the BCR^a^	[53]
*BANK1*	4q24	SLE, SSc, RA	BANK1	Scaffold protein involved in BCR signalling	[54]
*IL21*	4q27	SLE, PSO, CEL	IL-21	Cytokine, class switch recombination, plasma cell differentiation^a^	[55]
*BACH2*	6q15	SLE, AS, ATD, CEL, CD, MS, T1D, IBD, PSC	BACH2	Negative regulator of transcription^a^	[50]
*PRDM1-ATG5*	6q21	SLE, RA, CD	Blimp1	Differentiation and development of plasma cells^a^	[49]
*IKZF1*	7p12.2	SLE, CD	Ikaros	TF, differentiation, development, self-tolerance^a^	[53]
*BLK*	8p23-p22	SLE, SS, RA, SSc, pAPS	BLK	Tyrosine kinase, BCR signalling, development	[56]
*LYN*	8q12	SLE	Lyn	Tyrosine protein kinase, BCR signalling	[4, 57]
*CCL21*	9q13.3	RA	CCL21	Chemokine, germinal centre formation	[58]
*ETS1*	11q23.3	SLE	Ets1	TF, negative regulator of differentiation^a^	[59]
*CXCR5*	11q23.3	SS	CXCR5	Chemokine receptor, migration to B cell follicles^a^	[60]
*SLC15A4*	12q24.32	SLE	PTR4	Proton-coupled amino-acid transporter located in endolysosomes, autoantibody production^a^	[53, 61]
*ELF1*	13q13	SLE	Elf1	TF, binding the IgH enhancer^a^	[62]
*CSK*	15q24.1	SLE	Csk	Increases BCR-mediated activation of mature B cells^a^	[63]
*ITGAM*	16p11.2	SLE	CD11B	Regulation of BCR signalling^a^	[64]
*IRF8*	16q24.1	SLE	IRF8	TF, cell development^a^	[65]
*IKZF3*	17q21	SLE	Aiolos	TF, downregulation of the pre-BCR^a^	[65]
*CD40*	20q13.12	RA	CD40	Co-stimulatory molecule, promotes antibody production	[58]
*IKBKE*	1q32.1	SLE	IKKI	Phosphorylates IκBα^a^	[50]
*TNIP1*	5q33.1	SLE, SS, PS	NAF1	TNFAIP3 interacting protein^a^	[49, 60]
*TNFAIP3*	6q23	SLE, SS, RA, T1D UC, CEL, PSO	A20	Ubiquitination and negative signalling regulator ubiquitin editing enzyme^a^	[58, 60]
*PRKCB*	16p11.2	SLE	PRKCB1	Member of the PKC family, BCR-dependent NF-κB activation^a^	[66]
*UBE2L3*	22q11.21	SLE, CD, RA, CEL	UBE2L3	Ubiquinase, NFkB activation, plasmablast and plasma cell development^a^	[67]
*IRAK1/MECP2*	Xq28	SLE, RA	Irak1	TACI-dependent Ig class switching via MyD88^a^	[68, 69]
*REL*	2p16.1	RA	Rel	Survival and proliferation^a^	[70]
*TRAF1*	9q33.1	RA	Traf1	CD40 and TLR signalling^a^	[58]

The table lists polymorphic risk loci associated with the development of autoimmune diseases. The data are generated in GWAS and genes cited include those that encode proteins with known functions in B lymphocytes

*RA* rheumatoid arthritis, *SLE* systemic lupus erythematosus, *GT* Graves thyroiditis, *T1D* type 1 diabetes, *CEL* coeliac disease, *MS* multiple sclerosis, *CD* Crohn’s disease, *PSO* psoriasis, *UC* ulcerative colitis, *AS* ankylosing spondylitis, *ATD* autoimmune thyroid disease, *JIA* juvenile idiopathic arthritis, *AA* alopecia areata, *IBD* inflammatory bowel disease, *PSC* primary sclerosing cholangitis, *SS* Sjögren’s syndrome, *SSc* systemic sclerosis, *TF* transcription factor, *BCR* B cell receptor

^a^Not specific for B cells

The need for, and the ability to generate, a vast B cell repertoire to combat a universe of pathogens requires tolerance checkpoints and exquisite fine-tuning of B cell receptor (BCR) signalling to limit the emergence of pathogenic autoreactive B cells. Highly coordinated and integrated intracellular signalling transduced through the BCR and other co-stimulatory receptors, including innate pattern recognition receptors such as Toll-like receptors (TLRs), costimulatory/inhibitory molecules and cytokine receptors, are essential for regulating the outcome of BCR engagement by antigens. The available evidence indicates that minimal alterations in established thresholds of activating or inhibiting intracellular signalling can lead to a breakdown of immunological tolerance. This review provides a synopsis of current knowledge of signalling molecules and pathways involved in mediating and regulating B cell responses and how changes could lead to aggressive self-reactivity and autoimmune diseases.

## Signals Controlling B Cell Development and Functions

**Fig. 2:**
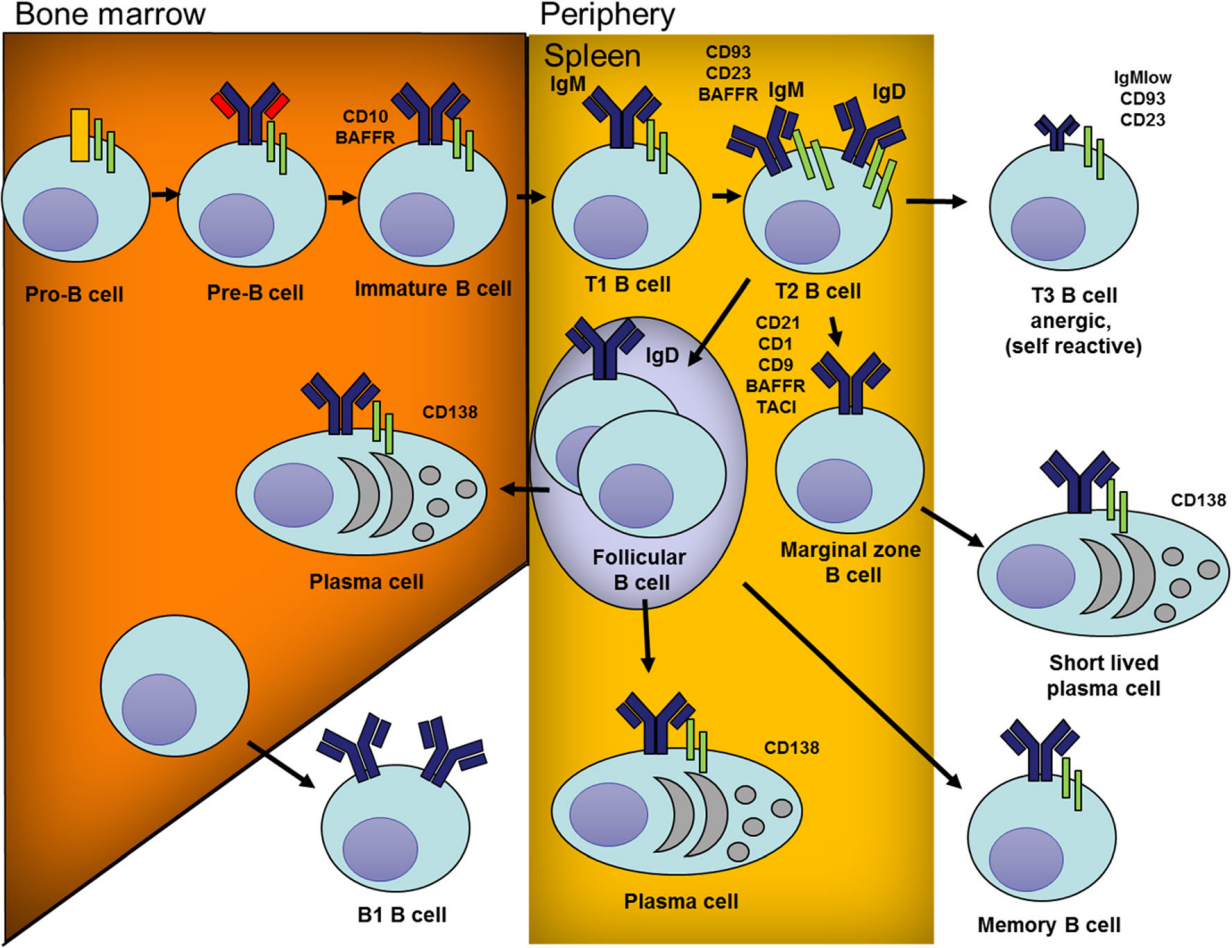
Pathways of B cell development and differentiation. B cells are generated from haematopoietic progenitor cells in the bone marrow. This process involves the expression of B lineage cell-specific proteins and the rearrangement of mini antibody V(D)J genes to generate the BCR repertoire. During the pro-B cell stage, antibody heavy chains are first generated by randomly rearranging and combining V, D and J mini genes. Pre-B cells express the pre-B cell antigen receptor (BCR) on the cell surface with the fully arranged heavy chain associated with the surrogate light chain (*red*). At later stages, light chain V and J mini genes are rearranged and a complete BCR is expressed in association with the Ig-α and Ig-β (*green*) subunits of the BCR complex. Immature B cells then undergo tolerance mechanisms with B cells recognizing self-protein undergoing light chain editing, apoptosis or functional inactivation (anergy). Surviving immature B cells then exit the bone marrow and migrate to secondary lymphoid organs where they develop into transitional (T) B cells. Transitional B cells can be subdivided into a number of developmental subsets. These include T1 B cells that express a high level of IgM and T2 B cells that express both IgM and IgD. These B cells undergo a range of tolerance checkpoint and cells that recognize self-antigens with high affinity are deleted. Cells with intermediate/low affinity to self-antigens and those that do not recognize self survive and circulate for about 3 weeks to survey the body for their target antigens. Transitional B cells develop into either marginal zone (MZ) B cells or follicular B cells. MZ B cells sample antigens and those that recognize antigens expand independently of T cell help. For their expansion, MZ B cells require TLR signalling to into short-lived plasma cells that produce antibodies with limited avidity for their target antigens. Follicular B cells are activated when they encounter their target antigens in the presence T cell help. Activated follicular B cells then migrate to B cell follicles and initiate somatic maturation in germinal centres. During this process, the cells proliferate, acquire somatic mutations, produce antibodies with higher avidity and class switch to IgG. Antigen-specific mature B cells then leave germinal centres and differentiate into either plasma cells or memory B cells. Plasma cells can either remain secondary lymphoid organs or travel to bone marrow to produce antibodies. B1 cells comprise a distinct subset of B cells that develop in the bone marrow and migrate to the periphery (peritoneal and pleural cavities in mice). B1 cells produce polyreactive IgM antibodies and partake in providing a first line of immunity against pathogens

The BCR repertoire for antigens is vast, generated through random recombination of germline V(D)J mini genes, to provide broad immunity against pathogens. However, an intrinsic feature of generating this vast repertoire is the randomness with which germline V(D)J mini genes are recombined. This leads, in up to 80% of newly generated B cells, to the generation of BCRs that recognize self (Fig. 2). There is, therefore, a necessity for emerging B cells to undergo tolerance in the bone marrow and also subsequently in the periphery for B cells that escape bone marrow tolerance or those that emerge as a result of mutations in secondary lymphoid organs.

Newly generated B cells first encounter self-antigens in the bone marrow, and their elimination or survival depends to a great extent on the strength with which their BCRs bind self-antigens and strength of the resulting intracellular signalling. Thus, recognition of self with high affinity initiates strong intracellular signalling, and as a result, B cells undergo receptor editing to replace nascent light chains that endow self-reactivity or, if this fails, apoptosis or anergy [84–87]. To facilitate B cell tolerance yet provide effective B cell immunity, intracellular signalling is regulated by highly refined thresholds. These thresholds regulate the magnitude and duration of intracellular B cell signalling and the outcome of BCR engagement by antigens. Subsequent to undergoing tolerance in the bone marrow, immature B cells migrate to the periphery where they will need tonic signalling for their transition to full maturity in readiness to respond to antigens. Signalling thresholds are also set for co-stimulatory receptors that modulate B cell responses following antigen recognition. These co-stimulatory receptors include CD40, TLR, BAFF and receptors for a range of other cytokines. Most of these signals involve the activation of phosphatidylinositol 3 kinase (PI3K) and the canonical pathway of NF-κB activation [88, 89].

### BCR-Mediated Signalling

#### Proximal BCR Signalling Initiates B Cell Development and Responses

The BCR associates with a heterodimer of signalling proteins, Ig-α and Ig-β (also known as CD79a and CD79b), to form the BCR complex. The cytoplasmic domains of both Ig-α and Ig-β have immunoreceptor tyrosine-based activation motifs (ITAMs). ITAMs initiate signalling when their tyrosine residues are phosphorylated following BCR engagement and translocation to lipid raft signalling domains that contain the Src family tyrosine kinase Lyn. Downstream, activation of ITAMs generates a docking site for spleen tyrosine kinase (Syk) recruitment and phosphorylation [89, 90]. The activation of Syk is fundamental for initiating signalling cascades leading to lymphocyte activation [90–92]. Substrates of activated tyrosine kinases are adaptor molecules that, in turn, recruit other kinases to the BCR complex. B cell linker protein (BLNK) is a Syk substrate with nine tyrosine residues that are rapidly phosphorylated following engagement of the BCR [92, 93]. Phosphorylated BLNK is recruited to the plasma membrane and this requires its association with CIN85. The BLNK-CIN85 complex then coordinates the recruitment of growth factor receptor-bound protein 2 (Grb2) and phosphoinositide phospholipase C gamma (PLCγ) [94], a process essential for B cell development and responses.

#### Defective Regulation of BCR-Mediated Signalling Leads to Aberrant B Cell Responses and Autoimmune Diseases

##### Src Family Tyrosine Kinase Lyn

Lyn is a key dual activity kinase. It initiates BCR-mediated signalling by phosphorylating Ig-α/Ig-β ITAMs but then regulates this signalling by phosphorylating immunoreceptor tyrosine-based inhibition motifs (ITIMs) in CD5, CD22 and FcγRIIB [95]. Thus, Lyn-deficient mice develop spontaneous lupus-like autoimmune disease, splenomegaly and glomerulonephritis and produce anti-dsDNA autoantibodies [96, 97]. In addition, BCR-mediated calcium (Ca^2+^) influx is enhanced in B lymphocytes in Lyn^−/−^, mice and there is accelerated class switching of anti-dsDNA and anti-RNA autoantibodies [97]. Interestingly, however, deletion of myeloid differentiation primary response gene 88 (MyD88) in Lyn^−/−^ mice, both globally or selectively in B lymphocytes, suppresses B cell activation and class switching of autoantibodies and ameliorates lupus disease [98]. This finding suggests that aberrant B cell responses in Lyn^−/−^ mice are likely to be influenced not only by BCR-mediated signalling bust also by signalling through TLRs.

In humans, there is evidence for reduced Lyn expression in B lymphocytes from patients with SLE and that this reduction impacts B cell responses. For example, B cells from Lyn-insufficient SLE patients produce IgG autoantibodies to dsDNA and disease-promoting cytokines in vitro [4]. The association between Lyn insufficiency and SLE is supported by genetic studies. Thus, single nucleotide polymorphism (SNP) analyses and genome-wide association studies have revealed that polymorphisms in *LYN*, as well as other Src family tyrosine kinases including *BLK*, are risk factors for susceptibility to SLE [56, 99].

##### CD45 Tyrosine Phosphatase

CD45 is a membrane protein tyrosine phosphatase that positively regulates Lyn activation by dephosphorylating a tyrosine residue at position 507 (Y-507). This causes a conformational change that exposes the catalytic domain of Lyn and promotes autophosphorylation of the positive regulatory tyrosine at position 396 (Y-396) [100]. In addition to Lyn, CD45 regulates the activation of other kinases, such as Janus kinases (JAKs) and, thus, influences cytokine signalling [101], Src kinases involved in cell adhesion [102], TLR signalling [103] and apoptosis [104]. Dysregulation of CD45, therefore, can affect multiple B lymphocyte functions leading to autoimmune-like diseases, but the precise impact of changes of each of the multiple pathways that CD45 regulates in promoting autoimmune disease remains unclear. In genetically engineered mice, a single nucleotide replacement in the dimerization wedge of the CD45 molecule was shown to lead to autoantibody production and the development of lupus-like disease [11]. However, it is established that CD45 also influences apoptosis and defects in its expression have been shown to promote lupus disease in Fas ligand-mutant (Fasl^*gld/gld*^) mice. In this setting, reduced CD45 expression enhanced B lymphocyte hyperactivity and auto-Ab production [105]. Furthermore, defects in CD45 regulation has been shown to affect B lymphocyte tolerance. For example, CD45^−/−^ mice and CD45^−/−^ B cell lines show reduced CD22 activation, SHP-1 recruitment, increased Syk activation [106] and Ca^2+^ influx [107]. Of note, however, is that loss of function mutations in sialic acid acetyl esterase (SIAE), which is required for the inhibitory function of CD22, has been shown to also create a significant risk for developing RA, T1D and SLE [108]. In hen egg lysozyme (HEL) transgenic mice, HEL induced tolerance in mature CD45^+/+^ B lymphocytes but led to the activation and accumulation of long-lived CD45^−/−^ HEL-reactive B lymphocytes [109].

Studies of B lymphocytes in patients with SLE in our laboratory revealed that Lyn insufficiency was associated with increased CD45 translocation to lipid raft signalling domains and, ultimately, to reduced cellular expression of this phosphatase [12]. The noted increase in the translocation of CD45 to lipid raft signalling domains is likely to be relevant to reduced Lyn expression since CD45 promotes Lyn activation, ultimately its degradation in the proteasome [4].

##### Downstream Kinases

Defects in the regulation of BCR-associated signalling molecules downstream of Lyn have also been reported and shown to promote aberrant B cell responses and autoimmune diseases. For example, defective regulation of the adaptor protein B cell adaptor protein with ankyrin repeats 1 (BANK1) which initiates BCR-mediated Ca^2+^ signalling after Lyn-mediated phosphorylation of inositol 1,4,5-trisphosphate receptor (IP3R) causes autoimmune-like disease in mice. Thus, while BCR-mediated Ca^2+^ influx was shown to be normal in Bank1^−/−^ primary B cells, this deficiency led to enhanced CD40-mediated proliferation, survival, increased Akt activation, and enhanced T-dependent antibody production and formation of germinal centres [110]. In humans, genetic studies have revealed that two variants of BANK1, R61H and A383T, are strongly associated with susceptibility to SLE [54]. The molecular basis for this association, however, remains to be determined. Nevertheless, the increase in CD40-mediated Akt activation in Bank1^−/−^ B cells suggests that the allelic variants may promote autoimmunity through affecting cognate B-T cell interactions.

More recent studies of B cells from patients with SLE carried out in our laboratory revealed that the extent of defects in intercellular signalling is more complex and extensive than previously thought with each of the many identified defects likely to impact different B cell responses and clinical symptoms differently [28]. For example, these studies identified defective regulation of PI3K, MAPK, cyclin-dependent kinase1 (CDK1) and PKC to varying degrees in B cells from patients with SLE compared with matched healthy controls. These studies also revealed that the activity of Rho, a serine/threonine kinase involved in cell motility, was reduced in B cells from patients with SLE. Although as stated above, the relevance of many of these defects remains to be determined, it is likely that reduced activity of Rho can lead to defective migration of B lymphocytes. In addition to the above defects, reduced activity of the cell cycle kinase ATR was noted in the SLE patients. ATR is involved in activating the DNA damage response pathway, which leads either to cell cycle arrest or apoptosis and is, therefore, a key checkpoint in regulating cell responses to DNA damage.

##### Protein Tyrosine Phosphatases

In addition to kinases that positively regulate BCR-mediated signalling, defects in phosphatases that control the activation of kinases downstream of Lyn have also been reported to be involved in promoting aberrant B cell responses in autoimmune diseases. For example, defects in LYP tyrosine phosphatase, which is encoded by the protein tyrosine phosphatase non-receptor 22 (*PTPN22*), were shown to be sufficient to promote systemic autoimmunity. GWAS studies also revealed that a SNP in *PTPN22*, 1858C/T that resulted in R620W amino acid substitution is associated with increased risk of SLE, TID and RA [111–113]. Interestingly, expression of the R619W LYP variant in B cells alone was shown to be sufficient to develop splenomegaly, spontaneous germinal centre formation, glomerulonephritis and anti-dsDNA autoantibody production [8].

### Calcium and Diacylglycerol Signalling

#### In Transcriptional Activation and B Cell Survival

The recruitment of PLCγ to the BCR signalling complex following engagement by antigens initiates phosphatidylinositol 4,5 biphosphate (PIP2) hydrolysis leading to the generation of inositol 1,4,5-triphosphate (IP3) and diacylglycerol (DAG) [94, 114]. DAG binds to the cysteine-rich domain of Ras/Rap guanyl-releasing protein and this activates rat sarcoma (Ras) and ras-related protein (Rap) GTPases, the serine/threonine protein kinase C (PKC) and protein kinase D (PKD). IP3, in contrast, binds to IP3 receptors on the endoplasmic reticulum (ER) to release Ca^2+^ from its stores and, thus, increase cytosolic Ca^2+^ concentration [114]. The depletion of Ca^2+^ stores in the ER is sensed by stromal interaction molecules 1 and 2 (STIM1 and STIM2). As a result, these proteins relocate to the ER-plasma membrane junction where they bind to the Ca^2+^-release activated channel (CRAC) protein Orail and/or canonical transient receptor potential 1 (TRPC1) channels allowing extracellular Ca^2+^ entry to increase its intracellular level. As a consequence, the sustained increase in intracellular Ca^2+^ triggers the activation of the Ca^2+^/calmodulin-dependent protein kinase kinases (CaMKKs), serine threonine kinases involved in the regulation of important cellular processes such as survival and cytoskeletal reorganization [115]. The BCR-induced increase in intracellular Ca^2+^ levels also activates calcineurin (also known as protein phosphatase 2B, PP2B), a protein phosphatase that controls intracellular localization of nuclear factor of activated T cells (NFAT) family of transcription factors [116, 117]. In resting B cells, NFATs are constitutively phosphorylated by casein kinase 1 and glycogen synthase kinase-3 (GSK3) and are sequestered in the cytosol as a result of binding to 14-3-3 proteins. The BCR-induced activation of calcineurin leads to dephosphorylation of NFATs, thus permitting their translocation to the nucleus. In the nucleus, NFATs form complexes with other transcription factors to regulate the transcription of target genes including IL-2, IL-4, IL-10, tumour-necrosis factor alpha (TNFα) and interferon gamma (IFNγ) [117, 118].

#### Defective Regulation of Ca^2+^ Signalling Promotes Defective B Cell Tolerance

Numerous studies have examined molecular mechanisms leading to aberrant Ca^2+^ signalling in B lymphocytes with special emphasis on how BCR and co-receptors CD19 and CD21 mediate PLCγ2-IP3-Ca^2+^ signalling. CD21-mediated Ca^2+^ signalling plays an important role in breaching B lymphocyte tolerance leading to autoantibody production [119]. Furthermore, changes in the regulation of Ca^2+^ signalling are recognized as a major signalling event that contributes to the loss of B lymphocyte tolerance. Noteworthy, and perhaps paradoxically, is that an elevation in the baseline level of Ca^2+^ and reduced BCR-mediated elevation have been noted in tolerized/anergic B cells [84, 120]. Elevated baseline level of Ca^2+^ is likely to be due to persistent but low level engagement of the BCR by self-antigens and a recognized characteristic of anergic B cells in experimental models [84, 121]. In response to antigen engagement, naïve B cells show a rapid increase in intracellular Ca^2+^ followed by a drop to reach a plateau within minutes. This plateau is similar to the basal level seen in anergic B cells and continues as long as the BCR is engaged by the antigen. Thus, anergic B cells represent the physiological equivalent of chronically antigen-stimulated naïve B cells. Modulating BCR-mediated mechanisms of Ca^2+^ signalling could, therefore, provide a potential therapeutic approach for treating autoimmune diseases. Indeed, treatment of B cells with 1,4-benzodiazepine Bz-423 which increases sensitivity to BCR engagement by causing sustained high level of Ca^2+^ promotes apoptosis [122]. Since hyperactivation and altered Ca^2+^ signalling are distinguishing features of autoreactive B cells, treatment with Bz-423 has been suggested to be a useful approach for eliminating autoreactive B cells in autoimmune diseases.

### Phosphatidylinositol 3 Kinase Signalling

#### PI3K Signalling in B Cell Development, Survival and Activation

PI3K signalling is important for B cell development, survival and activation. PI3Ks represent a family of lipid and protein kinases that function mainly through phosphorylation of phosphoinositide [123, 124]. Based on molecular structure and functions, PI3Ks are divided into four classes: I, II, III and IV. Members of class I PI3Ks are the ones whose altered activation is implicated in autoimmunity and inflammation. This class of PI3Ks is subdivided into two distinct subgroups, IA and IB. In mammals, the IA subgroup includes three members: PI3Kα, PI3Kβ and PI3Kδ [125]. All three kinase members of the IA subgroup are heterodimers consisting of p110 catalytic subunits (p110α, p110β and p110δ) and a regulatory subunit, usually referred to as p85 [125]. Subgroup IB, in contrast, consists of one catalytic subunit, p110γ, associated with either a p101 or p84 regulatory subunit [123]. These different PI3Ks function in different signalling pathways in lymphocytes with p110δ expression been restricted to haematopoietic cells. Upon receptor activation, PI3Ks phosphorylate PIP2 leading to the production of PIP3 [126]. The production of PIP3 requires recruitment of PI3Ks to the plasma membrane either through binding of the SH2 domain of their regulatory units to the phosphorylated tyrosine residues in receptor signalling complex domains and adaptors, or through direct recruitment by Ras. Signalling through PI3K is negatively regulated by the lipid phosphatase, SH2 domain-containing inositol phosphatase (SHIP) and phosphatase and tensin homologue deleted on chromosome ten (PTEN) [127]. Upon engagement of the BCR, CD19 recruits PI3K to the plasma membrane through binding of p85 to its tyrosine-phosphorylated cytoplasmic domain. In B lymphocytes, the B cell activating factor (BAFF) and low basal signalling by un-engaged BCR maintains low PIP3 levels [87]. The level of PIP3 increases dramatically following BCR engagement by antigens and co-stimulation through CD19, IL-4 receptor and/or TLRs. The recruitment and binding of the key downstream target of PI3K, Akt [also known as protein kinase B (PKB)], to the PIP3 through its pleckstrin homology (PH) domain causes conformational changes to Akt and, as a result, permits PIP3-dependent kinase 1 (PDK1)-mediated phosphorylation of Akt at threonine 304 within its catalytic domain. PDK1 has a PH domain that binds PIP3 and promotes its translocation to the plasma membrane to co-localize with Akt [128]. Once activated, Akt phosphorylates important downstream targets including Rheb GAP TSC2, FOX1/3 and Fox4A. Akt-induced phosphorylation of Ras homologue enriched in brain (Rheb) GAP TSC2 that leads to the accumulation of Rheb-GTP complex results in the activation of mammalian target of rapamycin complex 1 (mTORc1) [129]. The Faxo family of transcription factors is active and located in the nucleus in resting cells; however, when phosphorylated by Akt, they translocate to the cytosol where their transcriptional activities are terminated. Akt is, therefore, important for metabolism and cell survival in peripheral B lymphocytes [87]. Additionally, Akt/Foxo pathway plays a critical role in regulating the expression of recombinase activating genes (RAGs) that are responsible for antigen-receptor rearrangement in B cells [130]. When Akt is inactive in quiescent B lymphocytes, Foxo1, Foxo3 and Foxo4A drive transcription of genes encoding IL-7, an essential homeostatic cytokine for lymphocytes, as well as for Kruppel-like factor 2 (KLF2) transcription factor [131]. KLF2 directly regulates the expression of adhesion molecules and chemokine receptors responsible for controlling B lymphocyte entry into and exit from secondary lymphoid organs.

#### Dysregulated PI3K Signalling Alters Normal B Cell Development and Differentiation to Plasma Cells in Autoimmune Diseases

The involvement of aberrant PI3K signalling in the pathogenesis of autoimmune diseases is intriguing as all leukocytes express all members of class I PI3Ks. However, evidence for the involvement of defective regulation of PI3K signalling has mainly emerged from studying pathways involving PI3Kγ and PI3Kδ as these two class I PI3Ks are exclusively expressed in immune cells. In contrast to PI3Kα and PI3Kβ where ablation of their genes leads to embryonic lethality [132], *PI3Kγ*- and *PI3Kδ*-deficient mice are viable but are immunodeficient [133–137]. Furthermore, enhanced activity of either PI3Kγ or PI3Kδ has been implicated in promoting autoimmunity [138–140]. In murine models of lupus and in SLE patients, the activity of PI3K is increased [141]. The exact cause(s) and impact of enhanced PI3K activity on SLE and autoimmune diseases in general remains to be determined. However, PI3K promotes B cell survival and the generation of short-lived plasma cells and suppresses class switch recombination through activating Akt, which, in turn, represses Foxo transcription factors [142, 143]. Of note, is that PI3Kδ is the main PI3K family member that is involved in regulating B lymphocyte responses. Thus, mice lacking PI3Kδ show reduced development of pro-B to pre-B cells in the bone marrow and impaired responses of mature B cells [136, 144]. Additionally, PI3Kδ is involved in regulating marginal zone (MZ) and B-1 B cell responses including antibody production [145]. Interestingly, B cell development in PI3Kγ^−/−^δ^−/−^ mice is similar to PI3Kδ^−/−^ mice, whereas no defects are seen in B cell development in PI3Kγ^−/−^ mice [146]. These observations indicate that PI3Kγ does not play a notable role in B cell development. Indeed, genetically engineered mice expressing a catalytically inactive PI3Kδ manifest impaired BCR signalling and reduced IgM and IgG antibody production [144]. Similarly, heterozygous deletion of PI3Kδ diminishes autoantibody production, ameliorates nephritis and improves survival in Lyn-deficient mice that develop lupus-like disease. In contrast, mice expressing constitutively active PI3Kδ show a reduced ability to eliminate autoreactive B lymphocytes [140]. In addition to its involvement in regulating BCR-mediated signalling, PI3Kδ is involved in mediating inflammation triggered by the engagement of TLRs [147]. These observations suggest that targeting of PI3Kδ could be an attractive therapeutic option for treating patients with autoimmune diseases and chronic inflammation [148–150]. Of note in this respect is that studies using mouse models of lupus have shown that inhibiting PI3K blocked glomerulonephritis and extended survival [139].

In addition to direct evidence for the role of dysregulated PI3K signalling in promoting autoimmune diseases in mice, there is indirect evidence for its involvement in promoting disease in patients. For example, there is evidence for decreased expression of PTEN, a lipid phosphatase that negatively regulates PI3K signalling in B cell subsets, except in memory B cells, in patients with SLE [31]. Furthermore, the level of PTEN in B cells from patients with SLE is inversely related to disease activity. Decreased levels of PTEN also concur with the upregulation of microRNA (miR-7) that downregulates PTEN expression. These findings suggest that defective miR-7 regulation of PTEN could contribute to B cell hyperresponsiveness in SLE [31]. Functional screening of a microRNA library also revealed that another miR, miR-148a, is a potent regulator of B cell tolerance [151]. Furthermore, increased expression levels of miR-148a were reported in patients with lupus and also in lupus-prone mice [151]. Elevated miR-148a levels impair B cell tolerance through enhancing the survival of immature B cells following BCR engagement by self-antigens [151]. Molecular studies revealed that miR-148a functions by suppressing the expression of Gadd45α, PTEN and the pro-apoptotic protein Bim. Furthermore, increased expression of miR-148a leads to lethal autoimmune disease in a mouse model of lupus [151]. Using adoptive transfer of anergic B cells, a recent study revealed that continuous signalling through the inhibitory molecules SHP-1 and SHIP-1 was required to maintain B cell anergy. Furthermore, reducing signalling through either of these two signalling pathways leads to rapid B cell activation, proliferation and the generation of short-lived plasma cells [152].

### Ubiquitination-Regulated Signalling

#### Ubiquitination Regulation of BCR-Mediated Signalling and Antigen Processing

Ubiquitination is an important posttranslational modification process that regulates signal transduction through covalent attachment of ubiquitin (Ub) moieties, a 76-amino acid peptide, to targeted proteins. The process involves at least three enzymes, Ub-activating enzyme (E1) that activates Ub, Ub-conjugating enzyme (E2) and Ub ligase (E3). E3 enzymes, such as Cbl, catalyse ligation of the C-terminal residue of Ub to a lysine residue on the target protein [153]. Lysine residues K6, K11, K27, K29, K33, K48 and K63 of Ub can potentially form seven different types of linkages in branched poly Ub chains, whereas a linear form of the Ub chain can be formed when only one lysine in each Ub in involved in linkage formation [153, 154]. Mono ubiquitination promotes endocytic trafficking and DNA repair and the K48-linked Ub moieties tag proteins for degradation via the proteasomal system. In contrast, K63-linked and linearly linked Ub chains provide docking sites for downstream effectors and promote protein-protein interactions and signalling [155]. Ubiquitination can also be regulated through deubiquitinating enzymes, proteases that remove mono-ubiquitins and poly-ubiquitins from proteins. In this regard, A20 acts as a deubiquitinating as well as an ubiquitin-editing enzyme. A20 inhibits the activation of NF-κB. It also restricts apoptosis induced by TNFα [156]. The following section will review data on two key effector enzymes involved in the ubiquitination cycle, Cbl and A20, since there is an abundance of evidence for their involvement in autoimmunity.

In mammals, the Casitas B lineage lymphoma (Cbl) family of proteins has three members: c-Cbl, Cbl-b, and Cbl-3. c-Cbl and Cbl-b are expressed in B cells [157] and function as prominent substrates for tyrosine phosphorylation and regulators of the threshold of signalling [157–160]. c-Cbl effectively inhibits B cell responses through downregulating Syk kinase [161]. c-Cbl and Cbl-b interact with several BCR-associated signalling molecules such as PLCγ2, BLNK, PI3 kinase, Lyn, Vav and Syk [42, 162, 163]. Subsequent to binding to ITAMs, Syk is phosphorylated on tyrosine 323 and this creates a binding site for c-Cbl [164]. c-Cbl recruits components of the ubiquitin conjugation pathway and acts as an ubiquitin ligase [165]. Binding of c-Cbl results in Syk ubiquitination and downregulation of BCR signalling [164]. Apart from regulating BCR signalling, c-Cbl mediates BCR ubiquitination, a process crucial for facilitating antigen processing and presentation by B cells through the internalization of antigen-BCR complexes and guiding them to multi-vesicular body-like MIIC. In these multi-vesicular body-like MIICs, antigen-BCR complexes are processed into peptides and loaded onto MHC class II for presentation to T cells [166–169]. The recruitment of Cbl-b to clustered BCRs is also required for the entry of endocytosed BCRs into late endosomes. Recruitment of Cbl-b is also required for the entry of TLR9 into endosomes as has been noted after in vitro activation of TLR9 by BCR-captured antigens [170].

In contrast to Cbl, A20 is a widely expressed cytoplasmic protein that inhibits NF-κB activation and signalling downstream of interleukin-1 receptor (IL-1R), TNF receptor 1 (TNFR1), CD40 as well as signalling through innate-type receptors such as TLRs and NOD-like receptors (NLRs) [171–174]. In addition, A20 promotes cell survival through which it can regulate immune responses [174]. By destabilizing E2 enzymes, A20 can disrupt the interaction between E2 and E3 and, therefore, restrict ubiquitination of target proteins [175]. To achieve its critical biochemical functions, A20 interacts with key effectors including the receptor interacting kinase-1 (RIPK1), a key player in inflammation and cell death, E2, E3, ABIN-1 (ubiquitin sensors) and NEMO/IKKγ, a key player in NF-κB signalling [176–181]. Additionally, A20 binds directly to ubiquitin chains [177, 179] and modifies ubiquitinated protein substrates in multiple ways. For example, A20 cleaves poly-ubiquitin chains, thereby, exhibiting a deubiquitinating activity. In addition, A20 works with E1 and E2 proteins to build ubiquitin chains, thus displaying E3-like activity [171, 182]. Through its Ub-editing functions, A20 also removes K63-linked poly-ubiquitin chains from substrates and builds K48-linked ubiquitin chains [182].

#### Altered Ubiquitination in Defective in B Cell Tolerance

Inappropriate ubiquitination has been associated with the development of autoimmune diseases. A large body of evidence implicates defects in the level and regulation of Cbl and A20 in the pathogenesis of autoimmune diseases. Thus, Cbl-b-deficient mice develop autoimmune diseases and highlight a connection between Cbl-b-mediated protein degradation and the regulation of BCR signalling thresholds [158]. These mice produce high levels of autoantibodies to double-stranded DNA and develop signs of spontaneous lupus-like disease [158]. Another study revealed that Cbl-b-deficient mice had an enhanced susceptibility to develop experimental autoimmune encephalitis (EAE) [183]. B cells from Cbl-b-deficient mice showed an enhanced ability to proliferate in response to BCR and CD40 engagements [158]. The lowering of BCR thresholds caused by the loss of Cbl-b correlated with increased susceptibility to develop autoimmune disease.

Many signalling proteins associate with Cbl-b, including PLCγ, PI3K, Syk and the adaptor proteins Slp-76 and Vav. However, Cbl-b-deficient cells have a selective enhancement of Vav phosphorylation, indicating that Cbl-b is a negative regulator of Vav phosphorylation. Vav is a key guanine nucleotide exchange factor for the Rho family of GTP-binding proteins [184], and mice with B cell-specific ablation of c-Cbl and Cbl-b manifest lupus-like disease and have a significant increase in MZ and B1 B cell numbers [184]. Interestingly, however, c-Cbl/Cbl-b-deficient B cells were not hyperresponsive to BCR engagement, did not proliferate extensively nor produced antibodies but tolerance induction was impaired [42]. Apart from attenuated BLNK phosphorylation, these mutant B cells showed enhanced phosphorylation of BCR-proximal signalling proteins including Syk, PLCγ-2 and Vav and increased Ca^2+^ mobilization. These results, therefore, indicate that Cbl proteins regulate B cell tolerance possibly through fine-tuning of BCR-mediated signalling thresholds [42].

In contrast to Cbl proteins, as cited earlier, A20 is expressed in all cell types and regulates the canonical pathway of NF-κB activation and promote cell survival. The regulation of these signals by A20 is important for preventing autoimmune diseases and defects could lead to autoimmune inflammatory diseases. For example, A20-deficient mice were shown to develop multi-organ inflammation and perinatal lethality which prevented detailed studies of A20 functions in adult mice [173]. However, mice lacking A20 expression specifically in B cells provided better insights into how A20 regulates B cell development and functions. These mice spontaneously developed a lupus-like disease characterized by increased plasma cell and germinal centre B cell numbers, elevated levels of IgM and IgG autoantibodies and immunoglobulin deposits in the kidney [37–39]. The increase in germinal centre B cell numbers could be due to resistance to FAS-mediated apoptosis [39] and/or enhanced expression of NF-κB-dependent anti-apoptotic proteins including Bcl-X. Of note, however, is that these mice did not develop renal failure but severe nephritis when lupus-prone mice were used. Furthermore, heterozygous mice which expressed reduced levels of A20 specifically in B cells manifested increased numbers of germinal centre B cells and produced autoantibodies [38]. In addition to enhanced BCR-mediated signalling, A20-deficient mice were hyperresponsive to TLR and CD40 engagement. Furthermore, when stimulated, A20-deficient B cells produced higher levels of IL-6 compared with wild-type B cells [39]. Enhanced IL-6 production in A20-deficient B cells may account for the moderate increase in T cell numbers in mice lacking A20 expression in B cells [39].

In humans, GWAS and SNP analyses of *TNFAIP3*, the gene encoding A20, revealed a potential role for A20 in susceptibility to autoimmune diseases in humans (Table 2) [185]. Subsequent studies confirmed an association with a number of autoimmune diseases including SLE [186], RA [187], psoriasis [188], T1D [189, 190] and SSc [191, 192]. Since mice expressing low levels of A20 develop spontaneous inflammation and autoimmune diseases [37–39], *TNFAIP3* SNPs might affect its function or expression. Indeed, reduced A20 functions in patients with SLE were associated with a SNP in the coding region of *TNFAIP3* that caused a substitution in residue 127 from phenylalanine to cysteine. In contrast, reduced A20 level was associated with a SNP at the 3′ enhancer region of *TNFAIP3* [193]. Additionally, it was suggested that SNPs located outside of the coding regions of *TNFAIP3* may confer susceptibility to diseases by reducing A20 expression [194, 195]. Polymorphisms could also have prognostic and therapeutic values. Thus, *TNFAIP3* polymorphisms and altered A20 expression levels were associated with therapeutic responses to RA patient treated with anti-TNFα agents [196]. The association of *TNFAIP3* polymorphisms with lymphoma in patients with Sjögren’s also highlights the potential role of A20 in regulating B cell hyperactivity and malignant transformation leading to lymphomagenesis [197]. Moreover, the presence of certain *TNFAIP3* SNPs was associated with the risk of severe renal or haematological complications in patients with SLE [193].

The NF-κB-associated signalling cascade is regulated by an E2 enzyme, UBE2L3 (also called UBCH7). UBE2L3 participates in the ubiquitination of p53, c-Fos and the NF-κB precursor p105, and defects are associated with increased susceptibility to many autoimmune diseases including RA and SLE [198, 199]. A single haplotype spanning *UBE2L3*, rs140490, was associated with increased UBE2L3 expression in B cells and aligned across multiple autoimmune diseases. Additionally, the *UBE2L3* risk allele correlated with increased numbers of plasmablasts and plasma cells in patients with SLE suggesting a role for UBE2L3 in plasmablast and plasmacyte development [67, 200].

### Innate Immune Receptor-Mediated Signalling

#### Innate Immune Receptor-Mediated Signalling and B Cell Tolerance

Innate immune receptors, such as TLRs, are pattern recognition molecules that bind conserved pathogen-associated molecular patterns (PAMPs) on pathogens. Ten TLRs are expressed in human cells, whereas in murine cells, there are 13 such receptors. Naïve human B cells express TLR1, 2, 3, 4, 6, 7 and 9, while plasma cells only express TLR3 and 4 [201]. TLR7 and TLR9 are known to be able to directly influence B cell tolerance. Engagement of TLR2 and TLR4, in contrast, has been implicated in promoting autoimmune diseases in mice although there is no direct evidence to support how, or indeed if, these two receptors modulate B cell. TLR7 and TLR9 are intracellular receptors that bind their ligands in endosomes. TLR7 binds ssRNA while TLR9 binds CpG DNA in viruses and bacteria. Interestingly, these receptors can be stimulated in self-reactive B cells by RNA and/or DNA-containing immune complexes. The two receptors dimerize upon ligand binding and recruit the adaptor protein myeloid differentiation primary response gene 88 (MyD88). The IL-1 receptor-associated kinase 4 (IRAK4) binds to MyD88 and activates IRAK1 and IRAK2. The resulting signalling complexes initiate the activation of NF-κB, MAPK and IFN-regulatory factor 1 (IRF1) and IRF5 signalling pathways and regulate the production of pro-inflammatory cytokine [202–205].

#### Dysregulated Innate Immune Receptor-Mediated Signalling Promotes B Cell Autoreactivity

A key feature of immunological abnormality in patients with autoimmune diseases is the production of autoantibodies, such as autoantibodies with specificity for nuclear antigens including DNA and proteins. There is evidence that crosstalk between signalling mediated by the BCR and TLRs could play an important role in the loss of B cell tolerance to these antigens [206, 207]. For example, BCR engagement by nucleic acid associated with self-antigens facilitates trafficking to endosomal compartments where TLRs reside leading to their engagement and B cell activation [208, 209]. In this respect, both TLR7 and TLR9 which initiate MYD88-dependent signalling pathways have been implicated in the pathogenesis of animal models of lupus and the production of anti-nuclear autoantibodies. Indeed, deletion of the *TLR7* gene in lupus mice suppresses the production of autoantibodies to RNA-associated proteins and ameliorate systemic autoimmunity. Paradoxically, however, deletion of the *TLR9* gene abolishes anti-dsDNA and anti-chromatin autoantibody production but exacerbates clinical symptoms [210, 211]. Since both TLR7 and TLR9 are expressed in B cells and myeloid cells, it is unclear whether the phenotype seen in these mice could be attributed to the effect of the two receptors on myeloid and/or B cells. However, deletion of TLR7 in Wiskott-Aldrich syndrome protein (WASp) in mice inhibited systemic autoimmunity, whereas deletion of TLR9 promoted systemic autoimmunity which recapitulates the phenotype seen in *TLR7/9*-deficient lupus mice [212–214]. WASp is expressed in haematopoietic cells and is implicated in BCR- and TLR-mediated signalling. Mutations in the *WASp* gene in humans cause Wiskott-Aldrich syndrome, an X-linked recessive disease characterized by primary immunodeficiency and high levels of autoantibodies [215]. In contrast to attenuating T cell receptor (TCR)-mediated signalling, WASp-deficient B cells are hyperresponsive to both BCR and TLR engagement leading to enhanced signalling adequate to mediate autoimmune disease even in the autoimmune-resistant B6 mouse [216]. In addition, WASp-deficient B cells are capable of activating wild-type CD4^+^ T cells and inducing spontaneous germinal centre formation, glomerulonephritis and the production of class-switched autoantibodies in mixed bone marrow chimeras in mice [216]. These effects were all MyD88-dependent since deletion of MyD88 in B cells abrogated T cell activation and spontaneous germinal centre formation [98, 217–219]. The pivotal role of TLR7 signalling in the pathogenesis of lupus was confirmed in several mouse models with the Y-chromosome-linked genomic-modifier Yaa in which there is duplication of the *Tlr7* gene [220]. In Yaa mouse models, duplication of the *Tlr7* gene was reported to be the sole requirement for accelerated autoimmunity and that reduction of *Tlr7* gene dosage abolished the autoimmune phenotype. Furthermore, in *TLR7* transgenic mice, B cells preferentially homed to spontaneous germinal centres in competitive chimeras suggesting a key role for TLR7-expressing B cells in driving the formation of autoreactive germinal centres [221]. Of note, overexpression of soluble RNAase ameliorated autoimmunity in TLR7-transgenic mice suggesting an important role for RNA in the pathogenesis of disease in these mice [222]. In genetic studies in humans, SNPs within *Tlr7* and polymorphisms in genes encoding proteins and transcription factors downstream of TLR signalling, including TNFAIP3, TNIP1 and IRF5, associate with susceptibility to SLE [53, 223–226]. In addition, variants of SLC15A4, a histidine transporter involved in lysosomal TLR signalling, also associate with susceptibility to SLE. Furthermore, deletion of SLC15A4 in B cells limits autoimmunity in murine models of the disease [61]. Noteworthy in this respect is that humans deficient in either IRAK4 or MYD88, downstream effectors of TLR signalling, show increased autoreactivity within the naïve B cell compartment suggesting a pivotal role for TLR signalling in regulating tolerance in B cells [227, 228].

### Co-stimulatory Receptor-Mediated Signalling

#### Co-stimulatory Receptor Signalling and B Lymphocyte Responses

The outcome of BCR engagement is influenced by signalling generated through a number of co-stimulatory receptors including CD5, CD19, CD21, CD22, CD40, CD45, CD72 and FcγRIIB. Signalling through these molecules upregulate and/or downregulate BCR-mediated signalling to fine-tune B cell responses. Any imbalance, or dysregulation, in signalling mediated through these co-receptors can either mediate autoimmune responses, or limit the ability of the immune system to mount an effective humoral response.

One of the key co-receptors involved in modulating BCR-mediated signalling is CD19. The cytoplasmic domain of CD19 has nine tyrosine residues which, when phosphorylated, act as docking sites for SH2-containing adaptors and kinases including PI3Ks, Vav-family guanosine exchange factors (GEFs) and growth factor receptor-bound protein 2 (Grb2). The engagement of CD40 by its ligand, CD40L, in contrast, initiates signalling through TNFR-associated factors (TRAFs) leading to the activation of downstream signalling pathways including MAPKs and NF-κB.

The activation of BCR-mediated signalling is also regulated by protein tyrosine phosphatases (PTPs), some of which, such as CD45, play dual positive and negative roles as cited earlier. Cytoplasmic phosphatases are recruited to the BCR complex through ITIM-containing co-receptors, such as CD5, CD22 and the low-affinity Fcγ receptors, specifically FcγRIIB [229]. Altered expression and/or activation either of kinases or phosphatases can lead to defective BCR-mediated signalling which, in turn, alters B-lymphocyte responses [230–232].

Co-ligation of the BCR and the FcγRIIB by antigen-antibody complexes leads to tyrosine phosphorylation of ITIMs [233], which, in turn, recruit SHIP, a lipid phosphatase with specificity for 5′-phosphate of PIP3 [234] through SH2-domain-mediated binding. SHIP dephosphorylates PIP3 to produce PI(3,4)P2 and, thus, diminish BCR-mediated elevation of PIP3. B lymphocytes also express the siglec family member CD22, an ITIM-containing receptor which interacts with ligands carrying a 2–6-linked sialic acids [235]. CD22 modulates BCR signalling threshold and inhibits signalling by recruiting SHP-1, a tyrosine phosphatase.

CD72 is constitutively expressed on B cells at all stages of their development except on plasma cells. CD72 negatively regulates BCR-induced signalling by recruiting SHP-1 through its cytoplasmic ITIM motif [236]. CD72 plays an essential regulatory role in modulating BCR-mediated signalling in autoreactive B cells [237]. In anergic B cells, CD72 downregulates BCR-mediated signalling by limiting antigen-induced Ca^2+^ influx and the activation of NFATc1, NF-κB, MAPK and Akt. Noteworthy, CD72 associates with SHP-1 and Cbl-b, suggesting a role for SHP-1 and Cbl-b in CD72-mediated inhibitory effects on BCR-mediated signalling in anergic B cells. CD100, a ligand for CD72, can turn off the negative effect of CD72 by inhibiting the phosphorylation of CD72 and, consequently, disrupting the interaction between SHP-1 and CD72 [238, 239].

#### Dysregulated Co-stimulatory Receptor-Mediated Signalling Promotes Autoreactive B Cell Expansion and Autoantibody Production

Cognate interactions between B and T cells involving co-stimulatory receptors such as CD40 and CD40L are critical for thymus-dependent humoral immunity. Ligation of CD40 induces B cell proliferation, class switching and somatic mutations. In lupus disease, loss or blockade of cognate B-T cell interactions involving CD40-CD40L ameliorates disease and prolongs survival in the NZB/NZW F1 and MRL-*lpr* spontaneous models of lupus [240–242]. In addition, the use of agonist anti-CD40 antibodies inhibits apoptosis of rheumatoid factor (RF) precursor B cells in arthritic mice, while blockade of CD40-CD40L abolishes RF production in transgenic mice [243, 244]. Furthermore, *cd40l* gene-deficient mice or treatment of neonatal NOD mice with anti-CD40L antibodies suppresses autoimmune diabetes [245–248]. Moreover, treatment of EAE mice with anti-CD40L antibody improved disease [249, 250]. Preclinical assessment of anti-CD40 antibody in a model of multiple sclerosis (MS) in monkeys provided additional support for the importance of CD40-CD40L interaction in autoimmune diseases [251–253]. However, clinical trials of anti-CD40L in patients with lupus had mixed outcomes, and in addition, some patients developed thromboembolism [254–256].

As cited above, in addition to CD40, defective regulation of engagement or signalling through other co-stimulatory receptors such as CD5, CD22 and FcγRIIB can also promote autoimmune diseases. CD5 and CD22 negatively regulate BCR-mediated signalling through ITIMs in their intracellular domains and PTPs. The PTPs can have dual inhibitory and activating effects. In autoimmune diseases, there is substantive evidence that defects in the regulation of co-stimulatory receptors and associated PTPs promote lupus disease, both in animal models and in patients. For example, there is evidence for altered expression of CD22 and SHP-1 in patients with SLE [4, 6, 257]. In genetically engineered mice, deletion of *cd22*, *FcγRIIB* or *PTPN6*, which encodes SHP-1, leads to B lymphocyte hyperactivity, auto-Ab production and lupus-like disease [5, 96, 258]. However, it remains unclear whether defects in the regulation of these co-stimulatory receptors and associated PTPs in patients are inherent or result from the disease process or, indeed, if they have a causal relationship with the disease.

In addition to the role of dysregulated kinases and phosphatases that regulate proximal BCR signalling, defects in co-stimulatory receptors that regulate downstream signalling have been associated with the development of autoimmune diseases. For example, dysregulation of CD72 has been shown to promote autoimmune diseases. In anergic B cells, CD72 constitutively regulates BCR-mediated signalling and limits proliferation and survival through suppressing cyclin D2 expression and Rb phosphorylation, key regulators of the cell cycle. Indeed, CD72-deficient mice spontaneously produce autoantibodies and develop lupus-like disease. Furthermore, CD72-deficient B cells proliferate and survive when their BCRs are engaged by self-antigens. The proliferative response of anergic B cells to BCR engagement by self-antigens results in the loss of immunological self-tolerance, upregulation of cyclin D2 and Bcl-xL, proliferation and survival of autoreactive B cells [259]. In contrast to anergic B cells where calcineurin/NFAT and NF-κB signalling pathways are defective [260, 261], self-antigen binding to anergic CD72^−/−^ B cells leads to the activation of both calcineurin/NFAT and NF-κB [259]. Both calcineurin/NFAT and NF-κB are required for the induction of cyclin D2 [262, 263] and activation of MAPK and Akt, key regulators of cell cycle and survival.

### Cytokine-Mediated Signalling

#### Cytokine Signalling in B Cell Differentiation

Dynamic regulation of cytokine production and cytokine receptor expression is required for B cell development, differentiation and efficient immune responses. Cytokines are involved in cellular communications and signalling and initiate a wide range of effects including cell differentiation, proliferation and regulation. Cytokines involved in B cell differentiation and responses include interferons (IFNs), interleukins (ILs) and members of the TNF family of ligands and receptors. Almost 40 cytokine receptors are known to initiate intracellular signalling mostly through JAKs and signal transducers and activators of transcription (STATs) [264, 265]. In addition to activating JAKs and STATs, cytokines can also initiate other signalling pathways such as activating Ras and PI3Ks [266, 267]. The binding of cytokines to their receptors induces dimerization or polymerization of the receptors and this activates associated JAKs. Activated JAKs induce phosphorylation and homo- and hetero-dimerization of STATs. Dimerized STATs translocate to the nucleus where they induce transcription of their target genes. There are four JAKs (JAK1, JAK2, JAK3 and TYK2) and seven known STATs (STAT1, STAT2, STAT3, STAT4, STAT5a, STAT5b and STAT6). Receptors for type 1 IFN signal via JAK1 and Tyk2, whereas receptors for IL-12 and IL-23 signal through JAK2 and Tyk2. Receptors for IL-2, IL-4, IL-7, IL-9, IL-15 and IL-23 signal through JAK1 and JAK3, whereas IFNγ receptor signals via JAK1 and JAK2 [268].

BAFF, which is a member of the TNF family of ligands and receptors, is crucial for B cell survival and development [269, 270]. The cytokine can bind to three different receptors: BAFF-R, B cell maturation antigen (BCMA) and transmembrane activator and Ca^2+^ modulator and cyclophilin ligand interactor (TACI) [271, 272]. Binding of BAFF to BAFF-R plays a predominant role in B cell maturation and survival, and mice deficient in either BAFF or BAFF-R exhibit a B cell developmental block at the T2 stage of transitional B cell maturation. In contrast, B cells in BCMA- or TACI-deficient mice develop normally into mature cells [273]. BAFF-R is linked to TRAFs and signals through both the canonical and alternative NF-κB pathways as well as through MAPK and PI3K pathways [274, 275]. Activation of the NF-κB pathway by engagement of the BAFF-R rescues transitional B cells receiving BCR signals from apoptosis in response to engagement by self-antigens, possibly through increased transcription of anti-apoptotic proteins and posttranscriptional modifications of pro-apoptotic proteins [276–278]. Interestingly, recent studies have revealed that there is crosstalk between BAFF-R and BCR signals that can induce survival signals independent of NF-κB activation [279, 280]. Thus, BAFF promotes rapid phosphorylation of proximal BCR signalling involving Ig-α and Syk. Deletion of Syk impairs survival and renders B cells non-responsive to BAFF. Survival in this setting can partially be restored by ectopic activation of MAPK or PI3K suggesting that BAFF-R signalling is likely to facilitate BCR-induced survival through activation of MAPK and PI3K. In this respect, BCR and CD19 have been implicated in regulating BAFF-R levels [86, 273].

#### Altered Cytokine Profiles Impacts Intracellular B Cell Signalling and Responses

Studies over the last few years have suggested that B cells can be subdivided into effector subsets based on the profile of cytokines they produce. Thus, B cells have been subdivided, in a manner akin to the subdivision of Th1 and Th2 cells, into B effector 1 cells that produce IFNγ and IL-12 and B effector 2 cells that produce IL-2, IL-4 and IL-6. More recent studies identified another effector B cell subset, B-regulatory cells (Bregs), characterized by their ability to produce IL-10, TGFβ and IL-35 and with immunosuppressive functions [281]. However, the available evidence indicates that, in contrast to effector T cell subsets, effector B cell subsets do not fulfil requirements of classic immune lineages such as defining transcription factors and may also exhibit plasticity depending on their microenvironmental settings. Nevertheless, the evidence provides support for a differential profile of cytokine production in B cells in different pathophysiological conditions. For example, altered profiles of cytokine production have been implicated in aberrant B cell responses in autoimmune diseases. Furthermore, in addition to the role of cognate T-B cell interactions in diverging cytokine production in B cells, TLR co-engagement with CD40 has been shown to synergize in promoting IL-10 and IL-35 production [282–284]. Both cytokines have important regulatory functions including limiting the generation of autoreactive germinal centres [284]. Interestingly, IL-10 can have dual effects on autoimmune diseases: acting as a B cell stimulator and also suppressor of T cell activations [285]. In this respect, IL-10 impacts autoimmune disease pathology differently depending on which cell and/or mechanism drives a disease. For example, while the transfer of IL-10-producing Bregs drives Treg cell expansion and modulates arthritis in mice, treatment of SLE patients with IL-10-specific monoclonal antibodies ameliorates disease [286, 287]. This outcome is consistent with evidence showing that high levels of IL-10 correlate with lupus disease activity in patients [288]. Interestingly, however, there is also evidence for defective signalling that regulate IL-10 production by B cells in patients with SLE [281, 282]. Similar to IL-10, IL-35 production by B cells has been shown to have immune regulatory functions and essential for recovery from EAE in mice [283].

In addition to the differentiation of B cells to distinct effector subsets with different cytokine profiles that impact autoimmune diseases differently, altered regulation of cytokine signalling in B cells can promote or enhance autoimmune disease pathology. For example, lupus is associated with high levels of IFNα and INFγ production with both altering B lymphocyte responses and autoantibody isotype production [289–291]. IFNα lowers BCR activation thresholds and promotes B cell differentiation through activating IRF5 transcription factor [292]. Indeed, polymorphism in the *IRF5* gene has been associated with susceptibility to SLE [7]. Interestingly, excess production of IFNα can be induced by immune complexes suggesting a positive feedback circuit between IFNα and autoreactive B cells in lupus [290]. High levels of IFNγ, in contrast, enhance the production of complement-fixing IgG subclass of autoantibodies in lupus mice and promote lupus disease in patients with RA when treated with the cytokine [293]. Further evidence for the harmful role of excess IFNγ production in promoting B cell abnormalities and lupus diseases comes from studies in which deletion of IFNγ receptor in B cells abrogates spontaneous germinal centre formation, class switching of autoantibodies and nephritis in lupus-prone mice [294].

Studies in humans and mice have also revealed an important role for excess IL-21 production in promoting B cells to differentiate to plasma cells [295]. Thus, IL-21^−/−^ mice have a diminished ability to produce IgG1 in response to immunization, whereas transgenic mice with enhanced expression of IL-21 develop hypergammaglobulinemia [295]. In contrast, IL-21 blockade successfully ameliorates lupus symptoms in lupus-prone MRL mice. Furthermore, knocking out *il-21r* gene suppresses lupus manifestations in the BXSB Yaa mouse model [295].

Overproduction of other cytokines, such as BAFF, a cytokine crucial for peripheral B cell development, has also been implicated in promoting autoimmune diseases [269, 270]. As cited earlier, BAFF binds to BAFF-R, BCMA and TACI. BAFF-R plays a key role in B cell maturation and survival and excess BAFF production is noted in many autoimmune diseases including SLE, RA and MS [296]. Indeed, transgenic mice expressing a high level of BAFF (BAFF-Tg) display B cell hyperplasia and develop lupus-like disease [297]. Of note in this respect is that high levels of BAFF rescue low-affinity self-reactive transitional B cells from negative selection at tolerance checkpoints and allows them to become mature B cells [298, 299]. These observations suggest that signalling through BAFF-R synergizes with BCR-mediated signalling in autoreactive B cells to override tolerance in these cells and permit the generation of pathogenic autoreactive B cells. Interestingly, MyD88, essential for TLR signalling, is crucial for autoantibody production in BAFF-Tg mice, suggesting that there is an interplay between BAFF-R and TLR signalling in promoting autoimmune diseases [297].

In addition to overt changes in cytokine production and signalling abnormalities in B cells, subtle changes in the regulation of signalling pathways activated by cytokine binding can also promote humoral autoimmunity. For example, heterozygous mutations in the *stat3* gene leading to replacements of key amino acids, such as K392R, M394T and K658N, and enhanced STAT3 binding, or dimerization are associated with multi-organ autoimmunity, lymphoproliferation and hypogammaglobulinemia with terminal B cell maturational arrest [300]. Similarly, SNPs in *stat3* and *stat4* genes have been associated with autoimmune thyroid diseases [301]. However, the mechanism involved in promoting altered B cell responses in individuals with these polymorphisms and how B cells promote disease are yet to be determined.

It is noteworthy that several JAK inhibitors have been recently developed as new therapies for treating patients with inflammatory autoimmune diseases such as RA. Thus, tofacitinib that inhibits JAK1, JAK2, JAK3 and, to a lesser extent, TYK2 is used in the clinic for treating RA patients in many countries (Table 3). JAKs are a family of non-receptor tyrosine kinases that are critical for signalling through cytokine receptors. At present, a number of other JAK inhibitors are in clinical trials for treating patients with diseases including RA and SLE (Table 3).

**Table 3:** Studies, clinical trials and approved therapeutic targeting of cytokines and signalling pathways in B cells for treating autoimmune diseases

Targeted signalling molecule/pathway	Agent used	Structure of agent	Biological effects on B lymphocytes	Disease/status	Reference
JAK1/JAK2/JAK3 and to a lesser extent TYK2	Tofacitinib	Chemical inhibitor	Inhibits the JAK/STAT pathway and blocks cytokine signalling	Approved for treating RA in many countries but not yet in the EU	[302]
JAK1/JAK2	Baricitinib	Chemical inhibitor	Inhibits the JAK/STAT pathway and blocks cytokine signalling	RA in phase III clinical trials	[302]
JAK3	Decernotinib (VX-509)	Chemical inhibitor	Inhibits the JAK/STAT pathway and blocks cytokine signalling	RA in phase II clinical trials	[302]
Pan-JAK	Peficitinib (ASP015 K)	Chemical inhibitor	Inhibits the JAK/STAT pathway and blocks cytokine signalling	RA in phase II clinical trials	[302]
JAK1	Filgotinib (GLPG-0634)	Chemical inhibitor	Inhibits the JAK/STAT pathway and blocks cytokine signalling	RA in phase II clinical trials	[302]
JAK1	ABT-494	Chemical inhibitor	Inhibits the JAK/STAT pathway and blocks cytokine signalling	RA in phase II clinical trials	[302]
JAK1/JAK2	INCB039110	Chemical inhibitor	Inhibits the JAK/STAT pathway and blocks cytokine signalling	RA in phase II clinical trials	[302]
JAK/SYK	R333	Chemical inhibitor	Inhibits the JAK/STAT pathway and blocks cytokine signalling	Discoid lupus in phase II clinical trials	[303]
JAK1	GSK2586184	Chemical inhibitor	Inhibits the JAK/STAT pathway and blocks cytokine signalling	SLE in phase II clinical trials	[303]
JAK1	GLG0778	Chemical inhibitor	Inhibits the JAK/STAT pathway and blocks cytokine signalling	SLE in phase II clinical trials	[303]
SYK	Fostamatinib	Chemical inhibitor	Inhibits SYK and blocks BCR and FcγR signalling	Clinical trials concluded that it is effective in treating RA; however, its clinical application is precluded due to unexpected side effects	[304]
BLyS (BAFF)	Atacicept	Recombinant fusion protein (TACI-Ig)	Blocks BLyS/APRIL binding and reduces survival and the number of some B cell subsets	Reduced B cell and plasma cell numbers and SLE disease activity but phase II/III trial stopped due to low blood Ab levels and pneumonia	[305]
	Belimumab	Fully human monoclonal Ab (mAb)	Inhibits BLyS binding to membrane receptors; promotes apoptosis of B lymphocytes	Approved for treating SLE. However, patients with active lupus nephritis are excluded. Use for active lupus nephritis at phase III clinical trials. Sjögren’s syndrome phase III clinical trials	[305]
	Briobacept (BR3-Fc)	Recombinant fusion protein	Inhibits BLyS binding to its receptor and promotes apoptosis	SLE clinical trials did not show sufficient efficacy	[305]
	Blisibimod (AMG-623)	Peptide-Fc fusion protein with 4 BLyS binding domains	Inhibits BLyS binding to its receptors and promotes apoptosis	SLE clinical trial is in phase III	[305]
IL-6	Sirukumab	Fully human mAb	Reduces B lymphocyte proliferation and differentiation	Clinical trials concluded its effectiveness in inhibiting progression of joint damage and improved signs and symptoms of disease in RA	[306]
IL-6R	Tocilizumab	Humanized mAb	Blocks B lymphocyte differentiation and reduces Ab production	Clinical trials concluded its effectiveness as a therapy for treating early RA	[307]
IFNα	Rontalizumab	Humanized mAb	Inhibits B lymphocyte activation and Ab production	Clinical trials concluded its effectiveness in treating SLE	[307]
	Sifalimumab	Fully human mAb	Inhibits B lymphocyte activation and Ab production	Clinical trials concluded its effectiveness in treating SLE	[308]
TLR4	NI-0101	Humanized mAb	Inhibits signalling through TLR4	In phase I clinical trial for treating RA	[309]
TLR7/8/9	Chloroquine	Chemical TLR7/8/9 antagonist	Reduces endosomal acidification and inhibits signalling through TLR	A mainstay therapy for SLE	[308]
TLR7/8/9	IMO-8400	Chemical TLR7/8/9 antagonist	Inhibits signalling through TLR7/8/9	In phase I clinical trials for SLE	[308]
TLR7-RLR9	IMO-3100	Chemical TLR7/9 antagonist	Inhibits signalling through TLR7/9	SLE clinical trial is in phase I	[308]

The table summarizes available information on the use of therapeutic agents to target signalling pathways in B lymphocytes in clinical trials and in practice

## Conclusions

It is now established that B cells play a key role in initiating and driving pathogenesis in many autoimmune diseases including SLE, RA, MS and SSc beyond their role in producing autoantibodies. Pathogenic roles played by B cells depend on tolerance status of the cells, which co-receptors are co-engaged with the BCR, the cytokine milieu and, ultimately, the nature and extent of intracellular signalling generated within B cells. The outcome of BCR engagement by antigens, self or exogenous, is determined by optimal levels or engagement and activation of kinases and phosphatases that regulate the strength and duration of intracellular signalling. Therefore, abnormal or incongruous engagement/regulation of signalling proteins, co-receptors or cytokines/TLRs can override B cell tolerance and lead to autoimmune disease development. Indeed, the available evidence indicates that regulated and coordinated engagement of co-receptors and signalling pathways ensures immune response specificity and efficiency and prevents the development of autoimmune diseases. This article has provided an overview of some of the key B cell signalling pathways and how defects in these could impact pathophysiology (Fig. 1). The available functional evidence on how changes in the level or function of signalling proteins and pathways impact B cell responses provides molecular mechanisms for how GWAS and SNP association with diseases such as SLE, RA, MS and TID could predispose to these diseases (Table 2). Noteworthy is that even in healthy individuals, autoreactive B cells, perhaps with low affinity for self-antigens, persist in the naïve B cell repertoire, yet these fail to become high-affinity pathogenic B cells. Thus, defining the molecular mechanisms that constrain the activation and/or regulation of self-reactive B cells will help understanding disease mechanisms and could also pave the way for new therapeutic strategies in precision medicine. Although B cell depletion therapy has proved to be highly effective in treating a number of autoimmune diseases including RA, MS and SSc, total ablation of B cells carries its own risks including life-threatening infections. In this respect, some recent studies have revealed that treatment of lupus mice with inhibitors of Bruton’s tyrosine kinase (BTK) can ameliorate disease [310, 311]. Thus, targeting dysregulated signalling effectors associated with proximal or downstream of BCR, CD40, TLR or cytokine receptors could prove an effective therapeutic strategy. However, despite substantial progress in the past few years in defining the role of altered B cell signalling in autoimmune diseases many questions and challenges remain.

## Take-Home Points

- B lymphocytes are essential for effective immunity to pathogens, but they can also cause diseases through producing autoantibodies, disease-promoting cytokines and presenting antigens to autoreactive T lymphocytes.
- The potential of B lymphocyte to cause diseases is prevented by tolerance and by a tight control of intracellular signalling pathways.
- Defects in intracellular signalling, however, can occur and these lead to autoimmune, lymphoproliferative or immunodeficient diseases.
- Defects in proximal BCR signalling due to reduced Lyn and/or CD45 levels promote autoreactive B cell activation, class switching to IgG autoantibodies and disease.
- Defects in downstream BCR signalling, e.g. Ca^+2^ or diacylglycerol pathways, lead to increased activation of NFAT transcription factor, tolerance defects, B lymphocyte hyperactivity and autoimmune diseases. Defects in PI3K lead to apoptosis defects and the generation of short-lived autoreactive plasma cells.
- Defects in ubiquitination, which regulates signalling in B lymphocytes through controlling protein levels and transcription factors, enhance activation leading to enhanced B lymphocyte proliferation to BCR and CD40 engagements and autoantigen presentation to T lymphocytes and lead to autoimmune diseases.
- Defects in innate immune receptor signalling, such as TLRs, exaggerate defects in BCR signalling and enhance autoreactive B cell expansion and responses.
- Dysregulated expression of co-stimulatory receptors, stimulatory or inhibitory, including CD5, CD19, CD21, CD22, CD40, CD45, CD72 and FcγRIIB, leads to B lymphocyte hyperactivity, auto-Ab production and autoimmune diseases.
- Altered cytokine production, cytokine receptor expression and/or cytokine signalling help rescue low-affinity self-reactive B lymphocytes, their differentiation and IgG isotype autoantibody production.
- Defects in the level and activation of JAKs and STATs enhance B lymphocyte differentiation leading to multi-organ autoimmunity, lymphoproliferation or hypogammaglobulinemia.
